# Utilizing noncatalytic ACE2 protein mutant as a competitive inhibitor to treat SARS-CoV-2 infection

**DOI:** 10.3389/fimmu.2024.1365803

**Published:** 2024-04-05

**Authors:** Shengjiang Liu, Haifeng Chen, Xiangqun Chen, Ningguang Luo, Sameera Peraramelli, Xiaoming Gong, Mingwei John Zhang, Li Ou

**Affiliations:** Avirmax Biopharma Inc., Hayward, CA, United States

**Keywords:** COVID-19, SARS-CoV-2, ACE2, neutralization, protein engineering

## Abstract

**Introduction:**

Angiotensin converting-enzyme 2 (ACE2) is an enzyme catalyzing the conversion of angiotensin 2 into angiotensin 1-7. ACE2 also serves as the receptor of several coronaviruses, including SARS-CoV-1 and SARS-CoV-2. Therefore, ACE2 could be utilized as a therapeutic target for treating these coronaviruses, ideally lacking enzymatic function.

**Methods:**

Based on structural analysis, specific mutations were introduced to generate mutants of ACE2 and ACE2-Fc (fusion protein of ACE2 and Fc region of IgG1). The enzyme activity, binding affinity, and neutralization abilities were measured.

**Results and discussion:**

As predicted, five mutants (AMI081, AMI082, AMI083, AMI084, AMI090) have completely depleted ACE2 enzymatic activities. More importantly, enzyme-linked receptor-ligand assay (ELRLA) and surface plasmon resonance (SPR) results showed that 2 mutants (AMI082, AMI090) maintained binding activity to the viral spike proteins of SARS-CoV-1 and SARS-CoV-2. In An *in vitro* neutralization experiment using a pseudovirus, SARS-CoV-2 S1 spike protein-packed lentivirus particles, was also performed, showing that AMI082 and AMI090 significantly reduced GFP transgene expression. Further, *in vitro* virulent neutralization assays using SARS-CoV-2 (strain name: USA-WA1/2020) showed that AMI082 and AMI090 had remarkable inhibitory effects, indicated by comparable IC50 to wildtype ACE2 (5.33 µg/mL). In addition to the direct administration of mutant proteins, an alternative strategy for treating COVID-19 is through AAV delivery to achieve long-lasting effects. Therefore, AAV5 encoding AMI082 and AMI090 were packaged and transgene expression was assessed. In summary, these ACE2 mutants represent a novel approach to prevent or treat COVID-19 and other viruses with the same spike protein.

## 1 Introduction

Human angiotensin-converting enzyme 2 (ACE2) is widely expressed on cell surfaces of various tissues, including the colon, duodenum, gallbladder, small intestine, heart muscle, lung, and airway (1). ACE2 is an enzyme that catalyzes the removal of a C-terminal amino residue of angiotensin 2 into angiotensin 1-7 (2). ACE2 has multiple functions, including as a negative regulator of the renin-angiotensin system and a facilitator of amino acid transport (3). In addition, ACE2 is important in regulating the normal biological functions of many types of tissues/organs. For instance, ACE2 is implicated in cardiovascular diseases, gut dysbiosis, inflammation, lung diseases, diabetic cardiovascular complications, and kidney disorders (4).

ACE2 has also been identified as a specific receptor for β-coronavirus, e.g., severe respiratory syndrome (SARS) coronavirus (SARS-CoV-1) (5–7), and HCoV-NL63, an α-coronavirus (8). Recently, human ACE2 has been identified as the specific receptor for the causative agent of COVID-19, SARS-CoV-2 (9). The binding of the viral spike protein (S) to ACE2, the viral receptor, initiates a virus replication cycle, causing host cell damage and viral transmission. SARS-CoV-2 has caused millions of patient deaths worldwide, posing significant challenges for the healthcare system (10). Inhibition of the virus binding to its receptor represents a novel strategy to manage COVID-19 prevalence.

SARS-CoV 1 and 2 virions bind their receptors of the host cells, the ACE2 ectodomain through the viral envelope spike protein (S1) receptor-binding domain (RBD) (11). Then, viruses enter cytosol through an acid-dependent proteolytic cleavage of S protein by cathepsin, transmembrane serine protease 2 (TMPRSS2), or other proteases, followed by the fusion of virus and cell membranes (11). Viral genomic RNA (gRNA) is released from the nucleocapsid, and synthesis of replicase using a gRNA template occurs. After replication of viral gRNA, structural proteins are translated and translocated into the Golgi for assembly (12). During the entire process, ACE2 plays a critical role in the replication cycle of SARS-CoV-1, SARS-CoV-2, and HCoV-NL63 (8, 12). Since circulating ACE2 blocks SARS-CoV-1 and SARS-CoV-2 from binding to its receptor on the host cell surface, viral infection and disease can be prevented and treated (13). Notably, the enzymatic activity of ACE2 is not involved in the viral entry process.

To manage the COVID-19 pandemic, multiple approaches have been taken, including vaccines (14), antiviral drugs (15), and antibodies (16, 17). The challenges of the approaches reside in the low or no protection when mutations in viral spike proteins occur naturally during viral transmission. Since the discovery of ACE2 as a SARS-CoV-2 receptor, no mutations affecting virus binding affinity have been identified, indicating the potential of ACE2 as a stable and specific therapeutic target. Initial efforts have been made to use it as the virus decoy receptor for COVID-19 (6, 18, 19). There is one Phase I/II clinical trial testing repurposing ACE2 (originally for treating pulmonary arterial hypertension and acute respiratory distress syndrome) as COVID-19 treatment (Clinical-Trials.gov identifier: NCT04335136). Moreover, as shown in another clinical study, ACE2 treatment in severe COVID-19 patients achieved a significant reduction of viral load in the serum, nasal cavity, and lungs (20). However, once ACE2 is directly administrated to a subject, as a virus-receptor blocker, other functions of ACE2 are also introduced, which may cause unnecessary activity associated with the renin-angiotensin system. Previous studies have assessed the feasibility of using ACE2 mutants for treating COVID-19 (21–24). In this study, based on structural analysis of ACE2 protein, specific mutations were introduced to generate previously unidentified mutants with depleted enzyme activity but similar binding affinity to viral spike proteins. The deletion of enzyme activity could avoid any potential adverse effect when a therapeutic dose level of wild-type ACE2 protein is administrated. That simplifies the mechanism of action when it is applied to patients as a pure and generic SARS-CoV neutralization agent. Further experiments showed that some mutants effectively bind to the S1 protein of SARS-CoV-1 and SARS-CoV-2. Therefore, these mutants represent novel therapeutic targets for treating coronavirus-related diseases (e.g. COVID-19) when delivered as proteins or via AAV vectors.

## 2 Materials and methods

### 2.1 Plasmid construction

ACE2-Fc variants were made through PCR and subsequent annealing of PCR amplicons with the NEBuilder HiFi DNA Assembly Kit (New England Biolabs, Ipswich, MA). The sequence of primers used for cloning mutants is listed in **Supplementary Table 1**. All sequences were confirmed by sequencing.

### 2.2 Transient expression and purification of mutants from HEK293 cells

Human HEK293 cells were cultured in DMEM medium (Thermo Fisher) with 10% FBS (ATCC Manassas, VA) in a CO_2_ incubator at 37°C. For maintenance passage, cells were split at 1:10 twice a week. For transfection, cells were seeded on a 10-cm cell culture dish (Corning, NY) at 2 x 10^6^ cells/dish in 10 mL media overnight. Each transfection of ACE2-Fc variant plasmids was performed using 14 µg/dish DNA with Lipofectamine 3000 reagent (Invitrogen, Carlsbad, CA). All transfections were performed in triplicate in at least three independent experiments. ACE2-Fc variant proteins were determined by the SDS-PAGE.

### 2.3 ACE2 enzymatic assay

The ACE2 enzymatic assay is based on the hydrolysis of an intramolecularly quenched fluorogenic ACE2 substrate, in the presence or absence of ACE2-specific inhibitor MLN-4760 (Merck Millipore CalbiochemTM ACE2 inhibitor, MLN-4760), which is a highly potent ACE2 inhibition with an IC50 of 440 pM. The ACE2 enzyme assay was performed in an enzyme assay buffer (50 mM 2-(N- morpholino) ethanesulfonic acid, 300 mM NaCl, 10 µM ZnCl2, pH 6.81). The ACE2 fluorogenic substrate, Mca-Ala-Pro-Lys(Dnp)-OH (AnaSpec, cat. # 60757, San Jose, CA, USA), was dissolved in 1% NH4OH to 15 mM. The substrate solution was aliquoted at 10 µL per vial and stored at -80°C. Protease inhibitor N-ethylmaleimide (Millipore Sigma Cat. 34115-5GM, St Louis, MO, USA) was 100 mM in Milli Q water, and phenylmethylsulfonyl fluoride (PMSF) was 100 mM in 100% ethanol. MLN-4760 was prepared as 10 µM in Milli Q water. The assay buffer/substrate mix is made freshly. In a reaction mix of 100 µL, 70 µL of assay buffer/substrate mix was added. Wildtype ACE2-Fc (AMI080) and five mutant ACE2-Fc variant proteins (AMI081, AMI082, AMI083, AMI084 and AMI085) were purified as described previously. These assays were performed on a 96-well microtitration plate. For each protein, it was diluted in sterile phosphate-buffered saline (PBS, HyPureTM, GE Healthcare, Hyclone Laboratories, Logan, Utah) at a range of 500, 100, 20, 10, 5, and 2.5 ng/mL. All reagents and buffer were added, mixed thoroughly, and immediately sealed, and wrapped with aluminum foil. The plate was placed on a shaking platform at 140 rpm at ambient temperature for 16-20 hours. The plate was read for relative fluorescence unit (RFU) using a fluorometer, *f*max (Molecular Device, Sunnyvale, CA, USA) with an excitation wavelength of 355 nm and emission wavelength of 460 nm. The data was average of duplicate readings.

### 2.4 Enzyme-linked receptor-ligand assay and surface plasmon resonance

All buffers were made in sterile water or PBS (Cat: SH30529.03, GE Healthcare Life Science, Logan, Utah). Each well was coated with 50 μL of 10 nM or 20 nM of spike protein 1 (S1) of SARS-CoV-1, SARS-CoV-2, or MERS-CoV diluted in sodium carbonate buffer (50 mM NaCO_3_, NaHCO_3_, pH 9.6) in a 96-well microplate. The S1 proteins were purchased from Sino Biological (SARS-CoV-1 S1 cat# 40150-V08B1, SARS-CoV-2 S1 cat# 40591-V08H, MERS-CoV S1 protein cat#: 40069-V08H, Beijing, China). The microplate was then tightly sealed, incubated at 2-8°C for 12 hours, and washed with PBS (10 phosphate buffer, 150 mM NaCl, pH 7.2, 0.01% Tween 20, PBS-T) for 3 times, followed by blocking with blocking buffer (1% BSA in PBS-T) at 37°C for 2 hours. After washing, 50 μL of serially diluted ACE2 variant proteins (20, 10, 5, 2.5, 1.25, 0.63, and 0.313 nM) were added in duplicate each well. The plates were sealed and incubated at 37°C for 60 min. The plates were washed with PBS-T 3 times. Then, 50 μL of goat anti-human IgG Fc-biotin conjugate (Abcam cat. Ab98618, Cambridge, MA) diluted 1:10,000 in PBS-T-0.5% (w/v) BSA was added to each well, followed by incubation at 37°C for 60 min. The microplates were washed 3 times with PBS-T. Afterward, 1:15,000 di-luted streptavidin horseradish peroxidase (HRP) was added at 50 μL/well and incubated at 37°C for 60 min. The microplates were washed 3 times with PBS-T and 1 time with PBS to remove the remaining Tween 20. The reaction was developed with 100 μL/well of 1-StepTM ABTS substrate (Thermo Scientific REF 37615, Rockford, CA) at 37°C for 30 min and stopped with 50 μL/well of 2% (w/v) SDS. The plates were read at 410 nm using a VERSAmax Microplate Reader (Molecular Device, Sunnyvale, CA). 50% binding concentration was calculated as the concentration when 50% of the maximal A410 readout was based on the curve.

Wildtype ACE2-Fc and variants were bound to Sensor chip protein A (GE Healthcare now Cytiva, cat 29-1275-57, Uppsala, Sweden) at 5 µg/mL in PBS with 0.01% Tween 20 (PBS-T). SARS-CoV-1 and MERS-CoV were obtained from Sino Biologicals (Beijing, China). The S1 proteins were diluted in PBS-T at final concentrations of 200, 100, 50, 25, 12.5, 6.25, 3.13, and 1.56 nM. The program was operated as binding kinetics using a BiaCore 3000 instrument.

### 2.5 *In vitro* neutralization with pseudovirus and SARS-CoV-2

Briefly, a gelatin-coated 96-well plate was seeded with 1.5x10^4^ 293T-hACE2 cells (CMV-hACE2) per well and cultured at 37°C, 5% CO_2_, and 95% humidity overnight. ACE2-Fc variant proteins were diluted in PBS at 20, 10, 5, 2.5, 1.25, 0.625, and 0.313 µg/mL in a separate 96-well “setup” plate (60 µL each well). Each sample was tested in duplicates. The pseudovirus stock was diluted into approximately 1 million infectious forming units (IFU) per mL (10^6^ IFU/mL). The diluted pseudovirus solution of 60 µL was added to all wells. The plate was mixed thoroughly and incubated at 37°C for 1 hour. A 100 µL mixture from each well of the setup plate containing the antibody and virus dilutions was carefully added to replace the medium in corresponding wells of the HEK293T-hACE2 cells plate. Finally, Trans plus™ (Alstem, Cat# V050, Richmond, CA) was added to each well per vendor’s manual. The plate was incubated at 37°C for 48-60 hours before reading for fluorescence.

The virulent neutralization assays were performed at Southern Research Institute (2000 Ninth Avenue South, Birmingham, Alabama 35205). The proteins were produced by transfecting monolayer cultures of HEK293 cells with AMI080 (wildtype ACE2-Fc), AMI082, and AMI090 plasmids. AMI080, AMI02, and AMI090 were serially diluted in PBS and transferred into wells of an empty ECHO plate (stock plate). Compounds were diluted 2-fold by transferring 40 µL of each stock sample into an adjacent well containing 40 µL PBS. This process was repeated to create 8 more wells of serially diluted samples and ensure that each well contains a 2-fold diluted sample of the previous well. A 90 nL aliquot for each sample is dispensed into corresponding wells of assay-ready plates using an ECHO555 acoustic liquid handling system. The final assay concentration range was 200 to 0.01 µg/mL at 2-fold serial dilution. PBS was added to control wells to maintain a consistent assay concentration of 0.3% in all wells. Then, Vero E6 cells were grown in MEM supplemented with 10% HI FBS and harvested in MEM (1% Pen/Strep supplemented with 2% HI FBS) on the day of assay. Assay-ready plates were pre-drugged with test com-pounds, AMI080, AMI082, and AMI090, which were prepared in the BSL-2 lab by adding 5 μL PBS to each well. The plates and cells were then passed into the BSL-3 facility. Cells were batch inoculated with SARS CoV-2 (USA_WA1/2020; M.O.I. ~ 0.002), which results in a ~5% cell viability 72 hours post-infection. A 25μL aliquot of virus-inoculated cells (4,000 Vero E6 cells/well) was added to each well in columns 3-24 of the assay plates. The wells in columns 23-24 contained only virus-infected cells as the 0% CPE reduction controls. Before virus inoculation, a 25 μL aliquot of cells was added to columns 1-2 of each as 100% CPE reduction controls. After incubating plates at 37°C, 5% CO_2_, and 90% humidity for 72 hours, 30 μL of Cell Titer-Glo (Promega) was added to each well. Luminescence was read using a BMG CLARIOstar plate reader following incubation at room temperature for 10 min to measure cell viability. Plates were sealed with a clear cover and the surface was decontaminated before the luminescence reading. For all assays, the raw data from plate readers were imported into Activity Base where values are associated with compound IDs and test concentrations. For the anti-viral CPE reduction assay, raw signal values were converted to % CPE reduction by the following formula:

% CPE reduction = 100 x (test protein value – mean value infected cell controls)/(mean value uninfected cell controls – mean value infected cell controls).

EC50 values were calculated from a four-parameter logistic fit of data using the Xlfit module of ActivityBase with top and bottom constrained to 100 and 0%, respectively.

### 2.6 AAV vector production, purification, and quantification

AAV vectors were packaged and purified as previously described (25). The *Spodoptera frugiperda* (Sf9) derived insect cell line, Sf9-V432A cells were cultured in corning storage bottles at 28°C in ESF AF medium (Expression Systems), supplemented with 100 units/mL penicillin and 100 μg/mL streptomycin (Corning). The cells were split 1:4 once the cell density reached 7 x 10^6^ cells/mL for maintenance. Recombinant baculovirus (rBVs) were generated according to Invitrogen’s protocol (Carlsbad, CA). Briefly, the constructed plasmids were used to transform DH10Bac and recombinant bacmid DNAs were isolated. The bacmid DNAs were transfected into V432A cells to generate rBVs. The rBVs were quantified with QPCR. Sf9-V432A cells were cultured to 7 x 10^6^ cells/mL and diluted 1:1 with fresh ESF AF media. About 200 viruses per cell of rBV containing the designated rep-cap genes and 100 viruses per cell of rBV containing the DNA sequences encoding ACE2 variant proteins were added separately to infect the Sf9-V432A cells for 3 days at 28°C in a shaker incubator. The infected V432A cells were harvested by centrifugation at 3,000 rpm for 10 min. Cell pellets were lysed in Sf9 lysis buffer (50 mM Tris-HCl, pH7.8, 50 mM NaCl, 2 mM MgCl_2_, 1% Sarkosyl, 1% Triton X-100, and 140 units/mL Benzonase^®^, Millipore, Burlington, MA). Genomic DNA was digested by incubation at 37°C for one hour. At the end of incubation, sodium chloride was added to adjust the salt concentration of the lysate to about 1 M to further dissociate the AAV vectors from the cell matrix. Cell debris was removed by centrifugation at 8,000 rpm for 30 min. The cleared lysates were loaded onto the CsCl step-gradient and subjected to ultracentrifugation at 28,000 rpm for 20 hours in swing bucket rotors. The viral band was drawn through a syringe with an 18-gauge needle, loaded onto CsCl, and subjected to linear-ultracentrifugation at 65,000 rpm for 20 hours. Then, the viral band was drawn and passed through two PD-10 desalting columns (GE HealthCare) to remove the CsCl and detergents and at the same time ex-changed to Buffer B (1 x PBS, 0.1M Sodium Citrate, and 0.001% Pluronic F-68). QPCR was performed to determine the AAV vector genome copy numbers with ITR primers and probes as below: ITR-QPCR-F: 5’-GGAACCCCTAGTGATGGAGTT-3’; ITR-QPCR-R: 5’- CGGCCTCAGTGAGCGA-3’; ITR-FAM-2ITR-MGB: 5’- CAC-TCCCTCTCTGCGCGCTCG-3’.

### 2.7 SDS-PAGE and SimplyBlue-staining to assess the purity of AAV vectors

The AAV5 vectors were mixed with SDS-PAGE loading buffer (Invitrogen) and heated at 95°C for 5 min. The vectors were then loaded onto a 10% SDS-PAGE gel and run at 100 volts until the dye reached the bottom of the gel. The gel was stained according to the manufacturer’s protocol (Invitrogen).

### 2.8 Measurement of transgene expression from AAV vectors

HEK293 cells were seeded at 1.5x10^5^ cells/well in 24-well plates and cultured overnight in 0.5 mL DMEM with 10% FBS. Then, cells were rinsed with serum-free DMEM and transduced with AAV5 encoding ACE2-Fc at various titers in 0.5 mL serum-free DMEM with 20 µM etoposide. After overnight transduction, the inoculum was removed and replaced with 0.5 mL/well DMEM containing 10% FBS. After transduction for a total of 72 hours, cell media were collected, added with proteinase inhibitor, and stored at ≤-65°C. HEK293 cell media (supernatants) collected 48 hours from plasmid transfection or 72 hours from AAV5-ACE2 transduction were used for Western blot. A total volume of 30 μL of cell supernatants was mixed with 10 μL of 4 x loading buffer and loaded onto the NuPAGE 10% Tris-Glycine gels (Invitrogen) for electrophoresis. Proteins were subsequently transferred onto PVDF membranes using X Cell II™ Blot Module (Invitrogen, Carlsbad, CA, USA). Membranes were treated with casein blocker in PBS (Thermo Scientific, Waltham, MA, USA) for at least one hour at room temperature and probed with the goat anti-human IgG Fc antibody conjugated with biotin (Abcam, Cambridge, UK), followed by incubation with streptavidin conjugated with horseradish peroxidase (Abcam). Proteins were detected using the ECL™ Western blotting kit (Amersham), and photos were recorded with iBrightTM CL1500 Imaging System (Invitrogen).

## 3 Results

### 3.1 Structure-guided design, molecular cloning, expression, and purification of mutants

**Figure 1:**
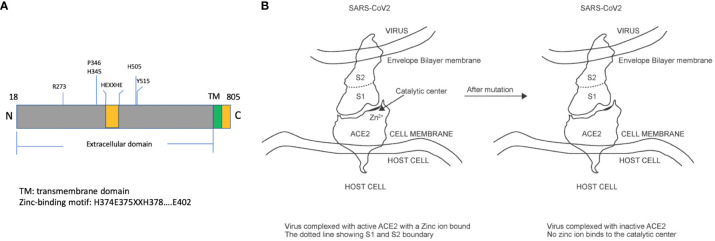
Illustration of human ACE2 protein structure and impacts of mutations on enzymatic activities. **(A)** The mature form of ACE2 protein (amino acid 18 to 805) includes a transmembrane domain and an extracellular domain, within which exists a zinc-binding motif responsible for catalytic activities. **(B)** The virus interacts with ACE2 through its S1 protein and manages to enter the cytosol through binding with ACE2. Some mutations in the catalytic center are expected to have no impact on the binding affinity.

ACE2 is a type I integral membrane carboxyl peptidase of 805 amino acids. The mature form (788 amino acids) contains an extracellular domain (725 amino acids), a short stretch of the transmembrane domain (21 amino acid residues), and an intracellular domain (44 amino acids) (26). Within the extracellular domain, there exists a “HEXXHE” metal ion-binding consensus sequence between H374 and E378. Also, E402 has been identified as the catalytic essential sequence (26) (**Figure 1A**). Mutation of the metal ion binding motif is expected to deplete the endopeptidase activity ACE2 completely but maintains its specificity and binding affinity for coronavirus (**Figure 1B**). In addition to the metal ion binding motif, several critical amino acid residues contribute to the enzymatic activity. The key residue arginine 273 (R273) of ACE2 plays a role in substrate recognition via a salt bridge and a hydrogen bond. H345 stabilizes the substrate-enzyme intermediate, while H505 also contributes significantly as the removal of H505 resulted in a 300-fold reduction of enzyme activity (27). Other residues important for the enzyme activity include P346 and H515. Based on the aforementioned descriptions, the complete abolishment of enzyme activity could be achieved by mutation of one and/or more than one of these residues, R273, H345, P346, H505, and H515. A large portion of COVID-19 patients have pre-existing cardiovascular diseases and are using angiotensin-converting-enzyme inhibitors and angiotensin II receptor blockers (28). The impacts of the Renin-Angiotensin-Aldosterone System (RAAS) blockade medicine on COVID-19 are unclear, with evidence showing RAAS blockade increased ACE2 expression and COVID-19 infection risk (29). In addition, it was observed that ACE2 treatment in COVID-19 patients led to a significant decrease in angiotensin II and a proportional increase in angiotensin 1–9 (20). Therefore, it is advisable to deplete the ACE2 enzymatic activity while using ACE2 as an anti-COVID-19 treatment.

**Figure 2:**
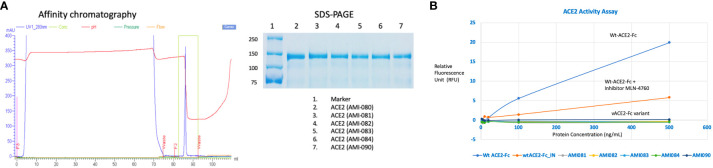
Determination of ACE2-Fc variants. **(A)** Variant characterization using affinity chromatography and SDS-PAGE. ACE2-ECD-Fc is also named as ACE2-Fc in the text as ECD stands for extracellular domain. **(B)** Enzyme activities of ACE2-Fc variants.

A series of ACE2-Fc variants were created by fusing to a human antibody heavy chain secretion signal peptide at the N-terminus and IgG1 Fc fragment at the C-terminus of the protein. The specific site mutation of the fusion protein was created by a rational design approach, which is based on structural analysis and an understanding of the physiochemical characteristics of amino acids. Once the ACE2 portion of the ACE2-Fc molecule binds to its specific virus, the Fc portion is expected to exert its biological function such as complement activation and Fc receptor-positive cells to attack the complex (30, 31). Also, ACE2-Fc has been shown to have improved pharmacokinetics and half-life in mice (32). ACE-Fc variants were generated through site-directed mutagenesis based on the 3D structure of ACE2 proteins and amino acid properties. The sequence information of these mutants is provided in **Table 1**. One mutation was recently reported by another study while this study is ongoing. G466D, when in combination with K465Q and E467K, led to increased binding affinity with SARS-CoV-2 (33). Mutants-encoding plasmids were transfected into HEK293 cells, followed by collection and purification of ACE2-Fc protein variants from transgene-expressing cells. Then, affinity chromatography and SDS-PAGE (**Figure 2A**) were performed, demonstrating the purity of these ACE2-Fc variant proteins.

**Table 1:** Sequence information of ACE2-Fc variants.

Sequence ID.	Amino acid mutation
AMI074	G466D
AMI080	ACE2-WT
AMI081	E402Q, G466D
AMI082	H374A, E402Q
AMI083	E375Q, E402Q
AMI084	H374A, E375Q, E402Q
AMI085	H374A, E375Q
AMI090	E402Q
AMI121	L79S, N330L, H374A, H378R, A386V, E402Q
AMI122	N330L, H374A, H378R, A386V, E402Q
AMI123	T27Y, L79S, N330F, H374A, A387L, E402Q
AMI124	T27Y, L79S, N330F, H374A, H378R, A387L, E402Q
AMI125	H374A, H378A, A387L, E402Q
AMI126	H374A, H378R, A387L, E402Q
AMI127	H374L, A387L, E402Q
AMI128	K26R, H374A, A387L, E402Q
AMI129	H374L, H378R, A387L, E402Q

### 3.2 Enzyme activity of mutants was depleted

The enzymatic activity of the affinity column chromatographic purified ACE2-Fc protein variants was measured as previously described (34). As shown in **Figure 2B**, wildtype ACE2-Fc protein was enzymatically active, as the relative fluorescence unit (RFU) increased with protein concentration. RFU was significantly reduced in the presence of ACE2 inhibitor MLN-4760, indicating the specificity of this enzyme assay. This enzyme assay was also highly reproducible, indicated by an inter-assay CV of 3.6% and intra-assay CV of 1-6%. Mutations in the Zinc-binding motif depleted ACE2 enzyme activity. For a better view of the catalytical activity of each ACE2-Fc variant protein, the enzyme reaction results are shown in **Table 2**.

**Table 2:** Enzymatic activities of ACE2-Fc variants.

Clone ID	Residue Mutated	Enzyme activity (RFU)@ 500 ng/mL
AMI080	ACE2-Fc wildtype	19.97 ± 0.11
AMI081	ACE2_E402Q-G466D-Fc	-0.59 ± 0.08
AMI082	ACE2_H374A-E402Q-Fc	-0.32 ± 0.03
AMI083	ACE2_E375_402Q-Fc	-0.55 ± 0.09
AMI084	ACE2_H374A-E375_402Q-Fc	-0.48 ± 0.06
AMI090	ACE2_E402Q-Fc	0.11 ± 0.04

### 3.3 Binding activity of mutants was maintained

**Figure 3:**
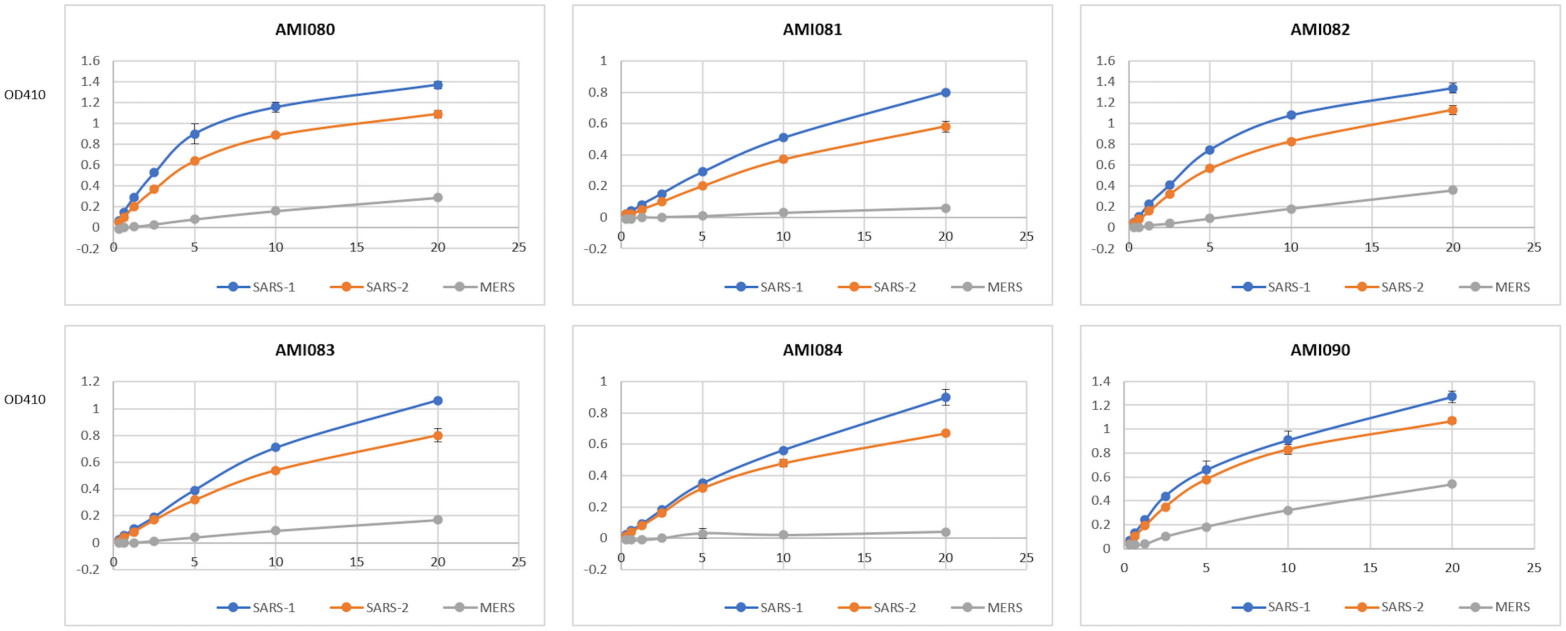
The binding ability of individual ACE2-Fc variants to three S1 proteins is determined by ELRLA. ACE2-Fc variants include AMI080, AMI081, AMI082, AMI083, AMI084, and AMI090. Viruses include SARS-CoV-1, SARS-CoV-2, and MERS-CoV.

The binding of 3 coronavirus spike proteins to each of the ACE2-Fc variant proteins was determined by ELRLA. SARS-CoV-2 spike protein bound to AMI080 (wildtype ACE2-Fc), AMI082 (H374A, E402Q), and AMI090 (E402Q) with a very similar binding profile (**Figure 3**). The estimated 50% binding concentration was about 11 nM for AMI080, AMI082, and AMI090. Similarly, the SARS-CoV-1 S1 spike protein bound the mutant AMI082 and AMI090 without significant difference from that of AMI080, with the 50% binding concentration at 7, 8, and 9 nM, respectively. AMI082 and AMI090 retaining the binding capacity to viral spike proteins demonstrated that H374 and E402 did not affect the binding of SARS-CoV-1 and SARS-CoV-2 S1 proteins to their cognate receptors on host cells. Surprisingly, AMI090 and AMI082 showed enhancement of binding to MERS-CoV S1 protein, indicating that AMI090 or AMI082 could be also used for blocking MERS-CoV infection. The other three mutants AMI081, AMI083, and AMI084 also showed significant binding to the spike proteins of SARS-CoV-1 and SARS-CoV-2.

**Figure 4:**
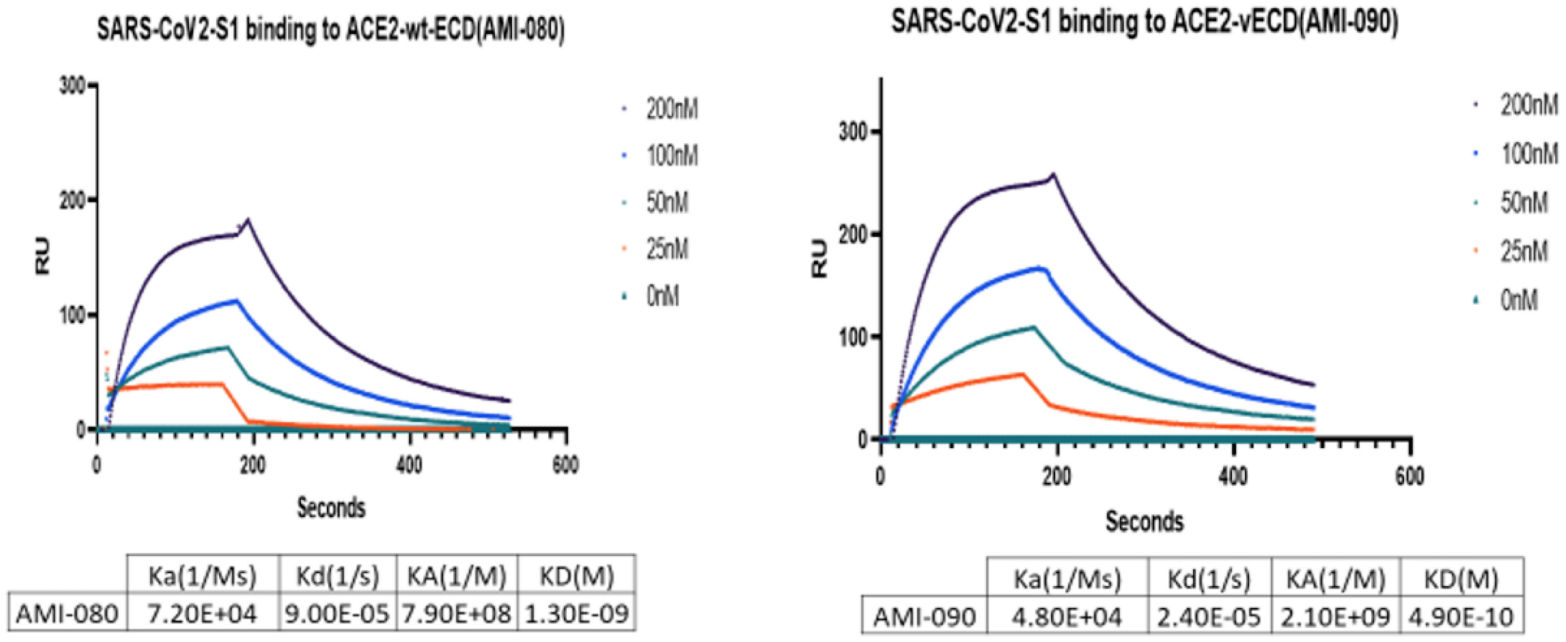
The binding affinity of ACE2-Fc variants determined by SPR. The binding affinity of AMI080 (left panel) and AMI090 (right panel) to SARS-CoV2 S1 protein.

To further determine the binding affinity of these ACE-Fc variant proteins to SARS-CoV-2 variants, purified proteins of the virus receptor binding domain (RBD) of SARS-CoV-2 B117 (N501Y) were tested. As shown in **Table 3**, AMI082 and AMI090 had similar binding affinity to the wildtype AMI080. In a qualitative binding assay, AMI080, AMI082, and AMI090 were evaluated for binding to various SARS-CoV-2 mutants. It was clearly shown in the test that the E484K variant of SARS-CoV-2 S1 RBD reacted to the ACE2-Fc proteins more strongly than other variants (**Table 4**). To further determine the binding capacity of wildtype ACE2-Fc and mutant proteins to the Spike 1 proteins of coronaviruses, the SPR method by BiaCore was also employed (**Figure 4**). The binding affinity (KD) of AMI080 was slightly higher than that of AMI090, indicating an increase in binding via Biacore assay.

**Table 3:** The binding affinity of ACE2-Fc variants to SARS-COV-2 B117 (N501Y) S1 receptor binding domain (RBD).

ACE2-Fc Variant	AMI080	AMI082	AMI090
Y_max_	0.024	1.146	2.294
EC_50_	1.0	1.3	1.1
KD (nM)	2.8	1.20	0.6

Y_max_ is the maximal binding of the assay, EC_50_ is the 50% binding of the maximal velocity, KD is the receptor-RBD interaction affinity, M(-1) or nM(-1), and df is KD difference of variant ACE2-Fc to that of the wildtype ACE2-Fc.

**Table 4:** Qualitative analysis of binding of ACE2-Fc variants to various SARS-CoV strains.

Variant Name	Mutation	Initial detected	Binding		
			AMI080	AMI082	AMI090
COVID-COV-2 S1 protein	Wildtype	Wuhan, China/2019	+	+	+
SARS-COV-2 S1 protein (D614G)	D614G	Europe	+	+	+
SARS-COV-2 S1 RBD (N501Y)	N501Y	UK	+	+	+
SARS-COV-2 S1 RBD (K417N)	K417N	India	+	+	+
SARS-COV-2 S1 RBD (E484K)	E484K	Africa	++++	++++	++++
SARS-COV-1 S1 protein	Wildtype	China/2003	+	+	+
MERS-CoV S1 protein	Wildtype	Saudi Arabia/2012	+	+	+

+ moderate binding was defined as A410 readout of 0.5-0.8, while +++ very strong binding as A410 readout of >3.0.

### 3.4 *In vitro* neutralization of SARS-CoV-2 by ACE2-Fc variants

**Figure 5:**
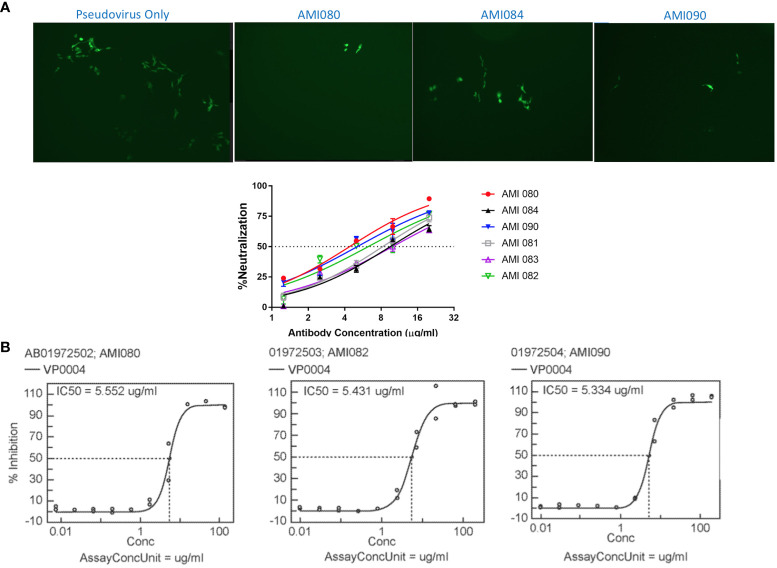
*In vitro* neutralization ability of ACE2-Fc variants determined by pseudovirus and SARS-CoV-2. **(A)** Neutralization assay using a lentiviral vector as pseudovirus. **(B)** Neutralization of SARS-CoV-2 by AMI080, AMI082, and AMI090.

The *in vitro* viral neutralization screening assay was performed using SARS-CoV-2 pseudovirus, a SARS-CoV-2 S1 lentiviral vector that expresses the green fluorescent protein (GFP) when it binds human ACE2 protein, the SARS-CoV-2 receptor on the cell surface of the stably transfected HEK293 cells (293T-hACE2). This is a safe and specific screening method for the evaluation of compounds, antibodies, or soluble receptors of the virus. 293T-hACE2 cells showed green fluorescent foci (GFF) in the absence of blocking or neutralization agents, and reduced GFF was seen when a specific neutralization reagent was present (**Figure 5A**). The 50% neutralization concentration was estimated at about 5 µg/mL for AMI080 (wildtype ACE2-Fc), AMI082, and AMI090. The other three constructs, AMI081, AMI083, and AMI084 were estimated to be at 10 µg/mL (**Figure 5A**).

The neutralization of SARS-CoV-2 by ACE2-Fc variant proteins (AMI082 and AMI090) was also performed using Vero6 cell culture infected with a virulent strain of SARS-CoV-2 (strain name: USA-WA1/2020, SARS-CoV-2). The analyzed data of SARS-CoV-2 (USA-WA1/2020) are shown in **Figure 5B**, which indicates the effective neutralization of SARS-CoV-2 achieved by AMI080, AMI082, and AMI090. The IC50 of the three proteins were 5.55, 5.43, and 5.33 μg/mL for AMI080, AMI082, and AMI090, respectively. To our surprise, the increased binding affinity of AMI090 to recombinant S1 proteins or RBD of SARV-CoV 2 showed similar neutralization against virulent SARS-CoV-2 (USA-WA1/2020) to that of the wildtype ACE2-Fc protein, AMI080.

### 3.5 AAV delivery of ACE2-Fc variants as a potential approach

AAV5 was selected for delivery of ACE2-Fc in this study because AAV5 is able to transduce airway epithelial cells (35). The purity of AAV vectors was determined by a SimplyBlue Staining assay. A typical gel pattern was obtained with expected VP1, VP2, and VP3 component levels (**Supplementary Figure 1**). Yields of AAV5 vectors were summarized in **Table 5**. AAV5 encoding ACE2-Fc variants were further evaluated for producing each construct protein using HEK293 cells. The Western blot image showed a single and sharp band of about 250 kDa for each construct using the non-reducing gel and no proteolysis was seen in the image. Together, these results demonstrated the feasibility of expressing ACE2-Fc variant proteins from AAV delivery, paving the way for future clinical development.

**Table 5:** Yields of AAV5 vectors determined through QPCR.

Vector name	AAV titer (vg/mL)	Total AAV Vol (mL)	Total Yield (vg)	Yield (vg/L)	Protein (µg/mL)
AMI089 (ACE2-Fc WT)	1.59E+13	4.6	7.33E+13	3.66E+14	286
AMI082 (H374A-E402Q)	2.09E+13	3.3	6.91E+13	2.30E+14	374
AMI083 (E375-402Q)	2.18E+13	4.3	9.38E+13	3.13E+14	419
AMI084 (H374A-E375-402Q)	1.67E+13	2	3.33E+13	1.11E+14	304
AMI085 (H374A-E375Q)	2.17E+13	5	1.08E+14	3.62E+14	541
AMI081 (E402Q-G466D)	2.76E+13	8	2.21E+14	1.10E+15	493
AMI090 (E402Q)	2.99E+13	12	3.58E+14	1.43E+15	494

## 4 Discussion

ACE2-Fc variant DNA can be cloned into a protein expression plasmid and transfected into mammalian cell lines, yeast, or other eukaryotic expression systems. The resultant cell line can be used for the production of the ACE2-Fc variant proteins via large-scale fermentation and a series of purification process steps. This product can be used for the urgent treatment of virus infection caused by SARS-CoV-1, SARS-CoV-2, MERS-CoV-1, or HcoV-NL63 infection, and maybe even possible for future emerging coronavirus using the same receptor for entry. Furthermore, the virus: ACE2-Fc variant proteins can be cleared through immune response pathways regulated by cells with IgG receptors, ultimately terminating the virus replication cycle. Once a virus particle enters the body, ACE2-Fc variant proteins function as a neutralization antibody, by binding viruses to form a protein-virus complex which can be eliminated by both inert and active immune cells. This is particularly valuable for elder people whose immune function is weak. Notably, all variants generated in this study were ACE2-Fc with mutations on ACE2. While the main goal of this study is to generate ACE2-Fc variants with therapeutic potential, admittedly, it will be interesting to include variants without Fc to have a better understanding of how fusion with Fc would affect the binding affinity. Prevention of coronavirus infection could be achieved by injecting a single dose of AAV5 encoding ACE2-Fc variants, which transduces many types of non-immune cells and produces sufficient levels of ACE2-Fc variant proteins *in vivo*. AAV can produce ACE2-Fc variant proteins for many years (36), thus this approach represents a novel method to provide long-lasting benefits.

Several studies have also attempted to utilize ACE2-Fc mutants as anti-COVID-19 therapy (24, 37–44). One study identified H378A, E402A, and R273A of ACE2-Fc proteins as potential candidates as they had no enzyme activities, but maintained binding affinity to SARS-CoV-2 (24). Another study tested multiple variants including H374A but not E402Q of ACE-Fc used in this study in a stringent K18-hACE2 mouse model (44). ACE2-Fc variant proteins successfully resolved lethal SARS-CoV-2 infection, in a prophylactic or therapeutic manner. In addition, this study also observed robust Fc functions, including antibody-dependent-cellular cytotoxicity, complement deposition, and phagocytosis. These results, including *in vivo* data, further supported the feasibility of the approach presented in the current study. The novelty of the current study resides in two matters. First, ACE2 E402Q is a novel mutation that has not been explored as anti-COVID-19 therapy. Second, AAV delivery of ACE2-Fc variants for long-lasting benefits versus one-time protein delivery. This has more advantages when considering the difficulty of manufacturing and purifying a large and relatively unstable protein (45) as ACE2-Fc (260 kD as a dimer) on a large scale. Notably, the binding affinity of AMI090 increased significantly compared with AMI080 (wildtype ACE2-Fc), while the neutralization ability is almost the same. This may be similar to the situation of total anti-body and neutralizing antibodies: not all binding leads to neutralization. Nevertheless, AMI090 still represents a promising therapeutic candidate. Notably, the neutralization ability of ACE2 mutants to more recent SARS-CoV-2 variants warrants further investigations.

Driving the balance of ACE2 activity toward Ang (1-7) and away from AngII represents a promising approach, while catalytically intact ACE2 is also being investigated. Nonetheless, our approach of creating non-catalytic ACE2 still has its own merits as the catalytic activity of ACE2, especially in continuous expression, poses a potential risk. Also, in this study, the feasibility of using AAV delivery to provide continuous ACE2 expression via a single dose was assessed. Therefore, the approach of creating non-catalytic ACE2 represents a promising alternative to current therapeutic options. In addition, mutants assessed in this study provide important information on ACE2 protein structure, which would be helpful for future mechanistic and therapeutic studies.

Although ACE2 is not the receptor of MERS-CoV, the binding affinity of ACE2 mutants was still assessed in this study as a recent study implicating the potential role of ACE2 in infection of two MERS-CoV-related viruses (46). Interestingly, it is the first time to report that the single mutation, E402Q, enables ACE2 to bind to MERS-CoV S1 protein and double mutations at H274A and E402Q MERS-CoV S1 binding capacity get weaker than E402Q alone, demonstrating the viral binding sites share some similarities between ACE2 and DPP4. It may be possible to select a common binding protein scaffold to bind both SARS-CoV and MERS-CoV spike protein molecules. Notably, a recent study reported TMEM106B as a receptor mediating ACE2-independent SARS-CoV-2 cell entry (47), it will be interesting to know whether ACE2 mutants promote the TMEM106B-dependent cell entry.

Despite the great potential, there remain several issues to be addressed before the clinical development of this approach. First, the immune response against AAV and ACE2-Fc could be elicited (48), resulting in adverse events and loss of transgene expression. Second, a large portion of AAV clinical trials (49) require the usage of immunosuppression. Since AAV vectors would take several weeks to achieve substantial transgene expression (50), other anti-COVID-19 treatments would be used due to the rapid progression of the disease. Then, the impact of immunosuppression on these anti-COVID-19 treatments should be carefully considered. Third, there also exists a potential risk of ACE2 overexpression toxicity. As shown in one NHP study, transgene expression alone from AAV vectors led to a loss of cells and animal deaths (51). Fourth, when delivered via AAV, how to minimize off-target expression as the AAV tropism is not limited to the target tissue, lung epithelial cells.

## Data availability statement

The original contributions presented in the study are included in the article/**Supplementary Material**. Further inquiries can be directed to the corresponding author.

## Ethics statement

Ethical approval was not required for the studies on humans in accordance with the local legislation and institutional requirements because only commercially available established cell lines were used. Ethical approval was not required for the studies on animals in accordance with the local legislation and institutional requirements because only commercially available established cell lines were used.

## Author contributions

SL: Conceptualization, Investigation, Writing – review & editing. HC: Conceptualization, Investigation, Writing – review & editing. XC: Data curation, Investigation, Writing – review & editing. NL: Data curation, Investigation, Writing – review & editing. SP: Data curation, Investigation, Writing – review & editing. XG: Data curation, Investigation, Writing – review & editing. MZ: Data curation, Investigation, Writing – review & editing. LO: Investigation, Writing – original draft, Writing – review & editing.
