# Supplementary material for: Utilizing noncatalytic ACE2 protein mutant as a competitive inhibitor to treat SARS-CoV-2 infection
Source: Front Immunol. 2024 Apr 5;15:1365803. doi: 10.3389/fimmu.2024.1365803 (PMC11032047; doi:10.3389/fimmu.2024.1365803)
Supplement: Supplementary file 1 [file DataSheet_1.docx]

**Table S1. Sequence information of primers used in molecular cloning of mutants.**

Primer ID DNA sequence

A024 5’-ATCCAGCCTCCGGACTCTAGAGTTAACTGGTAAGTTTAGT-3’

A056 5’-GTTGCCTTTACTTCTAGGCCTGCCGCCACCatgGAGTTCGGCCTGAGCTGGCTGTTCCT-3’

A074 5’-AACAGCTATGACCATG-3’

A098 5’-ATGTACGGGCCAGATATACGCGTTCGTTACATAACTTACGGTAAA-3’

A120 5’-TGATTATTGACTAGTATCTGCGTTACATAACTTACGGTAA-3’

A121 5’-ACTCcatGGTGGCGGCAGGCCTAGAAGTAAAGGCAACATC-3’

A122 5’-ATAAAGATATTTTATTTTCGAATTCTCAGC-3’

A123 5’-CTGTTCTACCAGAGCAGCCTGGCCA-3’

A124 5’-CTGGGAGAACAGCATGCTGACCGAC-3’

A125 5’-AGAGCATCAAGGTGAGAATCAGCCT-3’

A126 5’-CGGCCAGCCCGAGAACAACTACAAG-3’

A145 5’-TCGTGGGGCACGGGCTCCACCACGC-3’

A146 5’-GCGTGGTGGAGCCCGTGCCCCACGA-3’

A147 5’-TGGGGGGGAACAGGAACACGCTGGG-3’

A148 5’-GCGGCCCCAGCGTGTTCCTGTTCCC-3’

A156 5’-GAATCCTGATGTGCACCAAGGTGACCATGGACGACTTCC-3’

A157 5’-GGTGACCATGGACGACTTCCTGACCGCCCACGCCGAGATGGGCCACATC-3’

A158 5’-GCATGTTGAACAGCTTCT-3’

A159 5’-GACCATGGACGACTTCCTGACCGCCCACCACCAGATGGGCCACATCCAG-3’

A160 5’-GACCATGGACGACTTCCTGACCGCCCACGCCCAGATGGGCCACATCCAG-3’

A161 5’-CGCCAAGCTCTAGCTAGAGGTCGACGCGGCCGCTCGGTCCGCAC-3’

A162 5’-TTCCTGCTGAGAAACGGCGCCAACGAGGGCTTCCACcAGGCCGTGGGCG-3’

A163 5’-GGGGTCTCACGTTCATGTTC-3’

A169 5’-GAGATGGATGGTGTTCAAGGGCGAGATCCCCAAGGACCAG-3’

A170 5’-CTGGTCCTTGGGGATCTCGCCCTTGAACACCATCCATC-3’

A385 5’-CCGAAGGGCACGGTCAGGCTGTACA-3’

A386 5’-TGTACAGCCTGACCGTGCCCTTCGG-3’


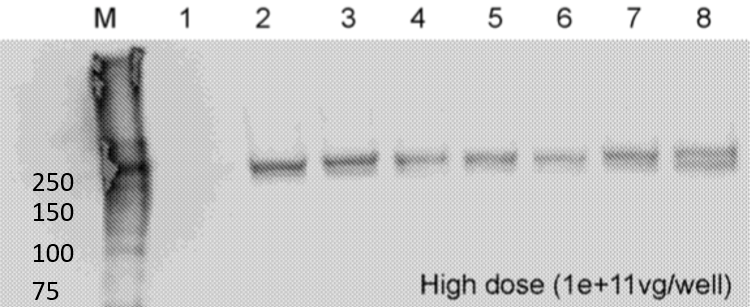


**Fig. S1. Western blot of ACE2-Fc variants expressed from AAV5 vectors.**

M, marker; 1, negative control; 2, AMI080; 3, AMI081; 4, AMI082; 5, AMI083; 6, AMI084; 7, AMI090; 7, AMI121.
